# Analysing Differences in Cognitive Health, Physical Fitness and Brain Activity in Older Women With and Without MCI

**DOI:** 10.3390/geriatrics10010025

**Published:** 2025-02-10

**Authors:** Wookwang Cheon, Jidong Tian, Jinkee Park

**Affiliations:** 1 Department of Physical Education, Keimyung University, Daegu 42601, Republic of Korea; wk11106@kmu.ac.kr (W.C.); jidong578@gmail.com (J.T.); 2 Department of Physical Education, Uiduk University, Gyeongju 38004, Republic of Korea

**Keywords:** old women, MCI, cognitive health, physical fitness, brain activity

## Abstract

The purpose of this study is analyse the differences in cognitive health, geriatric fitness, and brain activity between female elderly people with and without mild cognitive impairment (MCI) to contribute to the development of strategies for the prevention and management of MCI. The study included 56 female elderly residents of G city, and the participants were divided into an MCI group (32) and a non-MCI group (24). Cognitive health was assessed by an MMSE, and geriatric physical fitness was measured by various indicators such as strength, flexibility, and balance ability. Brain activity was measured by EEG(Electroencephalogram) to record concentration, stress, and left and right brain activity. Data were processed using independent samples *t*-tests and multiple regression analyses. The results showed that the MCI group was older, had more chronic diseases, and had a lower MMSE scores compared to the non-MCI group. In geriatric fitness measures, the non-MCI group had higher scores in handgrip strength and balance ability. In brain activity analyses, the MCI group had higher workloads and left brain activity than the non-MCI group, but there was no significant correlation with overall cognitive health. Regression analyses showed that, among the elderly physical fitness variables, vigour had a significant effect on cognitive health, suggesting that physical robustness may enhance cognitive reserve. The MCI group had lower cognitive health and physical fitness compared to the non-MCI group and showed some differences in brain activity. In particular, handgrip strength had a significant effect on cognitive health, suggesting that an exercise programme focused on strength training may be an effective intervention for the prevention and management of MCI.

## 1. Introduction

The rapid transition to an ageing society is increasingly highlighting the importance of health issues associated with cognitive decline, such as mild cognitive impairment (MCI). MCI is an intermediate stage of cognitive decline with a high probability of progression to dementia, making it a key issue in the management of cognitive health, particularly in older adults [1,2]. In fact, research suggests that older adults with MCI are more than 10 times more likely to develop dementia than the general population [3], further emphasising the need to develop strategies for early diagnosis and management of MCI [4]. Studies have repeatedly reported that MCI is strongly associated with cognitive decline, with MCI patients scoring lower than non-MCI patients on tests of cognitive function [5], and that cognitive decline may influence the rate of progression of MCI and the transition to dementia [6].

Studies exploring the relationship between brain activity and MCI suggest that activation in specific areas of the brain is associated with cognitive function. In particular, functional brain imaging (fMRI) studies have reported that people with MCI show abnormal activation patterns in certain brain regions when performing cognitive tasks [7]. However, it is not yet clear how effective this activation actually is in protecting or enhancing cognitive function [8]. In addition, studies that have examined the relationship between MCI and structural changes in the brain have found that people with MCI experience atrophy in certain regions of the brain and that these changes in brain structure are strongly associated with cognitive decline [9]. A multidisciplinary approach is needed to comprehensively understand these structural and functional changes in the brain, and it is particularly important to use neuroimaging studies to clarify the link between MCI and progression to dementia [10].

Aerobic exercise is known to play an important role in protecting brain function by improving cardiovascular function and increasing blood flow to the brain [11]. This effect is particularly pronounced in certain areas of the brain, such as the hippocampus, which is well known for its role in memory and learning [12]. Studies have shown that aerobic exercise increases the size of the hippocampus and improves memory, suggesting that aerobic exercise may contribute significantly to maintaining cognitive function beyond just physical fitness [13]. Physical activity may also play a positive role in the long-term maintenance of brain health by reducing stress and inflammatory responses and promoting neurogenesis, and these effects may have important implications for preventing the progression to MCI (mild cognitive impairment), particularly in older adults [14]. In addition to aerobic exercise, strength and flexibility training can also have a positive impact on cognitive function [15]. Strength training can improve quality of life and prevent falls by preventing muscle loss, maintaining balance and mobility, especially in older adults, indicating that taking care of physical health may have an indirect effect on cognitive health [16]. Balance ability is a complex process that involves the body and nervous system working together and has emerged as an important factor in maintaining cognitive function, particularly in older adults. Interventions that combine balance training with cognitive training help to improve attention spans and the ability to handle multiple tasks, and this approach may be effective in slowing or preventing cognitive decline in older adults [17].

Research into the effects of exercise on cognitive function is still in its infancy, with conflicting results making it difficult to draw consistent conclusions [18]. Some studies have reported no positive effects of certain forms of exercise on cognitive function, suggesting that systematic, long-term studies are needed to clarify the relationship between physical activity and cognitive function [19]. Such studies will provide useful data for public health policy formulation, with important implications for improving the quality of life of older adults, preventing dementia, and maintaining cognitive health. Therefore, future research should focus on gaining a deeper understanding of the effects of physical activity and developing practical interventions to slow or prevent the progression of MCI. In particular, a multidimensional approach incorporating demographic, physical, and cognitive factors is needed for early detection and management of MCI, and studies indicate that clear analyses of the interactions between factors are required [20,21].

Therefore, the purpose of this study was to analyse the differences in demographic variables, cognitive health, brain activity, and geriatric fitness between community-dwelling female older adults with and without mild cognitive impairment to provide comprehensive data on the maintenance of cognitive health in older adults with MCI and to analyse the interactions between these variables to contribute to the development of prevention and management strategies for MCI.

## 2. Method

### 2.1. Subjects

Fifty-six elderly women (MCI elderly women: 34, non-MCI elderly women: 22) living in the city of G were selected as the subjects of this study. The purpose of the study was fully explained and consent was obtained. The study is exempt from IRB review as it involves minimal risk to participants, does not include vulnerable populations, and meets the criteria under Article 15(2) of the Bioethics and Safety Act and Article 13 of its Enforcement Regulations. The characteristics of the study subjects are shown in Table 1.

### 2.2. Research Procedures

The study participants were recruited through welfare facilities and physical education facilities for the elderly located in City G. After explaining the purpose and procedures of the study and seeking their cooperation, the study participants were selected from the facilities that cooperated with the study. The inclusion criteria were limited to those who had no major trauma or surgery in the last 6 months, and the purpose of the study, procedures, expected benefits and risks, and privacy policy were explained to the participants; written consent was also obtained.

Data collection consisted of measuring participants’ demographic characteristics, dementia progression, physical activity, cognitive health, brain activity, and physical fitness. Demographic information was collected via a questionnaire, cognitive health was assessed via an MMSE, and brain activity was measured using EEG equipment. In addition, geriatric physical fitness was assessed through a variety of measures, including muscle strength (grip strength), flexibility, balance, aerobic capacity, gait, and muscle performance. All procedures were carried out with the comfort and privacy of the subjects as a priority.

### 2.3. Methods

#### 2.3.1. Cognitive Health

Cognitive health was assessed using the Mini-Mental State Examination (MMSE). Developed in 1975 by Folstein et al. [22], the Mini-Mental State Examination was modified and adapted by Lee et al. (1997) [23]. The 1989 MMSE-K consists of orientation in time and place (10), memory (3), recall (3), calculation and concentration (5), understanding and judgement (2), and language (7), with a total score of 30 [24]. Participants with MMSE scores of 24 or higher were classified as non-MCI, while those with scores below 24 were classified as MCI.

#### 2.3.2. Body Composition

Body composition was measured using the InBody 720, a bioelectrical impedance analysis (BIA) device. Participants were instructed to avoid eating or drinking for at least 2 h prior to the measurement and to remove any metallic items, such as jewellery, that could interfere with the device’s readings. The measurement was conducted with the participants standing barefoot on the device while holding the hand electrodes. The InBody 720 provided detailed measurements of body composition, including skeletal muscle mass, body fat mass, basal metabolic rate (BMR), body mass index (BMI), and waist–hip ratio (WHR). To ensure consistency and accuracy, all measurements were performed in the morning under standard conditions.

#### 2.3.3. Physical Fitness

Physical fitness was measured in terms of muscle strength (grip strength), flexibility (shoulder mobility), equilibrium (Single-Leg Stance Test with eyes open), aerobic capacity (step test), gait (Time Up to Go: TUG), and muscular performance (chair sit-up test).

- Grip strength was measured using a digital hand dynamometer. Participants were instructed to stand upright with their arms by their sides and squeeze the dynamometer with maximum effort using their dominant and non-dominant hands alternately. Each hand was tested twice, and the highest value for each hand was recorded in kilogrammes (kg). Adequate rest was provided between trials to prevent fatigue.
- Shoulder mobility was assessed using the back scratch test. Participants were asked to reach one hand over their shoulder and the other hand up the middle of their back as far as possible. The distance between the middle fingers (positive for overlap, negative for gap) was measured using a ruler. Each participant performed the test twice, and the best result was recorded.
- Balance ability was evaluated with the Single-Leg Stance Test. Participants were instructed to stand on one leg while keeping the other leg bent at the knee and their eyes open, focusing on a fixed point. The duration for which they could maintain this position without losing balance was recorded, up to a maximum of 60 s. The test was performed for each leg, and the best result was used for analysis.
- The step test involved participants stepping up and down on a 30 cm high platform at a consistent pace for 3 min. After completing the test, their heart rate was measured to determine recovery ability.
- The Time Up to Go (TUG) test measured participants’ mobility and gait efficiency. Participants were asked to rise from a seated position in a chair, walk a distance of 3 m, turn around, walk back, and sit down again. The time taken to complete the task was recorded using a stopwatch. The test was performed twice, and the fastest time was recorded.
- The muscular performance sit-up test was conducted to assess muscular endurance. Participants were instructed to sit on a chair with their arms crossed over their chest. Upon the start signal, they were asked to stand up completely from the chair and sit back down as quickly as possible. The number of times they could perform this movement within 30 s was recorded, ensuring that each repetition was performed fully and correctly for an accurate assessment.

#### 2.3.4. Brain Activity

The EEG test consisted of a sequence of evoked EEG tests which measure brain waves as participants solve spatial perception and memory tasks. Brain activity was measured using the BES-2000 (Brain Trainer Association, Seoul, Republic of Korea), a device specifically designed to evaluate cognitive function. The measurement utilised two frontal electrodes positioned on the Fp1 and Fp2 sites of the prefrontal cortex to record brain activity. Brain utilisation skills were assessed through the Brain Test™, which includes tasks targeting spatial recognition and memory. The Brain Test™ measures and analyses subcategory indicators such as cognitive strength, cognitive speed, concentration, brain stress, and left and right brain activity.

To ensure normal measurement results, participants were instructed to maintain a stable physical and mental state before testing, as factors such as beverages affecting heart rate, visual and auditory stimuli, emotional excitement, and recent exercise could influence outcomes. The detailed variables of brain activity are shown in Table 2.

### 2.4. Statistical Analysis

The statistical processing of this study was performed using an SPSS 27.0 win statistical programme to calculate the Mean and Standard Deviation of all measures. An independent sample *t*-test was used to test the difference between each measure with and without MCI. Pearson correlation was performed to determine the correlation between cognitive health, geriatric physical fitness, and brain activity, and a multiple regression analysis was performed to analyse the effects of geriatric physical fitness and brain activity on cognitive health. All statistical significance levels were set at *p* < 0.05.

## 3. Results

In this study, we analysed the differences between each item with and without MCI.

### 3.1. Differences in Demographics and Cognitive Health with and Without MCI

Differences in the demographic characteristics and cognitive health of those with and without MCI were analysed, as shown in Table 3. Older adults with MCI were significantly [t(40.99) = −4.044, *p* < 0.001] older than non-MCI older adults. Non-MCI seniors had a significantly [t(41.34) = 2.547, *p* < 0.05] higher cost of living than MCI seniors. MCI seniors had significantly [t(41.24) = −2.424, *p* < 0.05] more chronic diseases than non-MCI seniors. Non-MCI seniors had significantly [t(53.87) = 4.899, *p* < 0.001] higher cognitive health than MCI seniors. However, there were no significant differences (*p* > 0.05) in education, housing type, household size, and marital status.

### 3.2. Differences in Body Composition Between with and Without MCI

Differences in the body compositions of those with and without MCI were analysed, as shown in Table 4. There were no significant differences (*p* > 0.05) in body composition for height, weight, BMI, BMR, skeletal muscle mass, body fat mass, and WHR.

### 3.3. Differences in Brain Activity Between with and Without MCI

**Figure 1:**
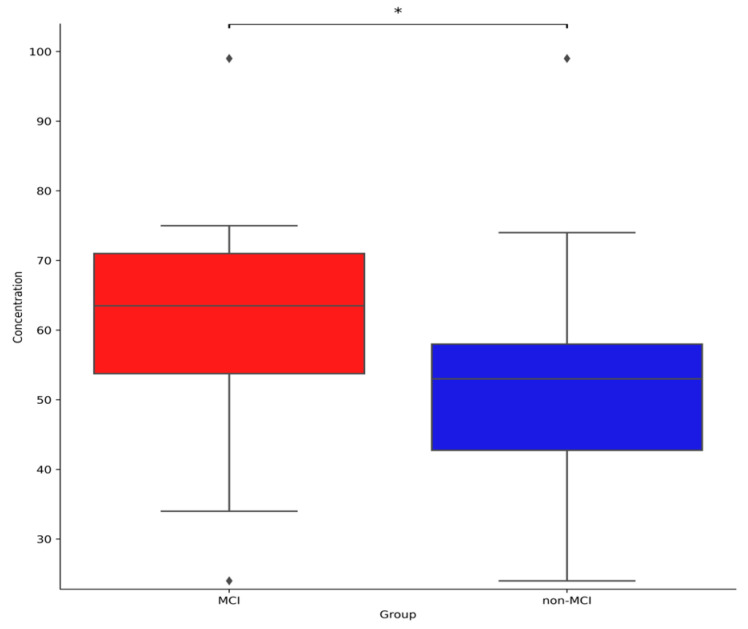
Differences in concentration between with and without MCI. * Indicates that the score was significantly different between the two groups (*p* < 0.05).

The results of analysing the differences in brain activity between MCI and non-MCI patients are shown in Table 5 and Figure 1. Concentration was significantly [t(53.48) = −2.514, *p* < 0.05] higher in MCI elderly patients than non-MCI elderly patients. On the other hand, there were no significant differences (*p* > 0.05) in Instant Memory, Judgement Time, Mental Workload, L Brain Activity, and R Brain Activity.

### 3.4. Differences in Physical Fitness in Patients With and Without MCI

**Figure 2:**
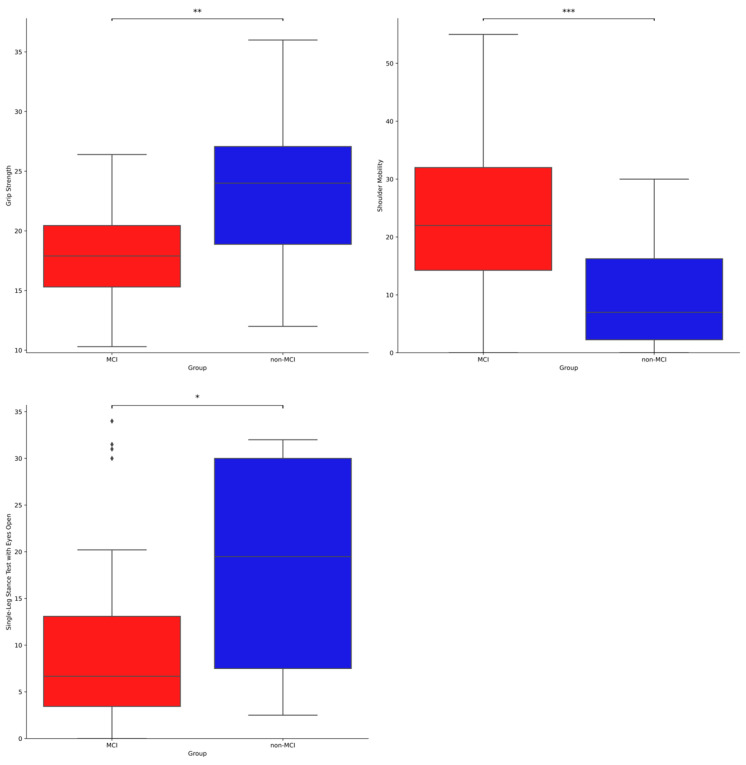
Differences in physical fitness between with and without MCI. * Indicates that the score was significantly different between the two groups (* *p* < 0.05, ** *p* < 0.01, *** *p* < 0.001).

Table 6 and Figure 2 show the results of analysing the differences in physical strength between the elderly with and without MCI. Grip Strength was significantly [t(36.22) = 3.145, *p* < 0.01] higher in non-MCI elderly patients than in MCI elderly patients. Single-Leg Stance Test with Eyes Open results were significantly [t(49.43) = 2.630, *p* < 0.05] higher in non-MCI seniors than in MCI seniors. Shoulder Mobility was significantly [t(53.98) = −4.197, *p* < 0.001] higher in MCI elderly patients than in non-MCI elderly patients. However, there were no significant differences (*p* > 0.05) in the Sit-Up Test, Step Test, and TUG.

### 3.5. Correlation Between Cognitive Health, Brain Activity and Older Adult Fitness

**Figure 3:**
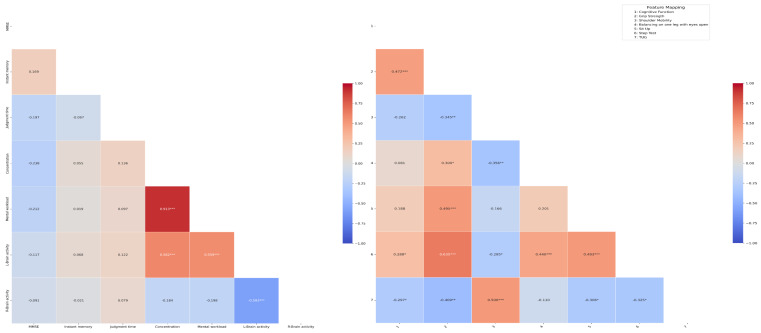
Correlation between cognitive health, brain activity and older adult fitness. * Indicates that the score was significantly different between the two groups (* *p* < 0.05, ** *p* < 0.01, *** *p* < 0.001).

The correlation between cognitive health, brain activity, and elderly physical fitness is shown in Figure 3. Concentration and Mental Workload (0.913 ***), L Brain Activity (0.582 ***), Mental Workload and L Brain Activity (0.559 ***), cognitive health and Grip Strength (0.472 ***), Step Test (0.288 *), Grip Strength and Single-Leg Stance Test with Eyes Open (0. 300 *), Sit-Up Test (0.495 ***) and Step Test (0.635 ***), Shoulder Mobility and TUG (0.500 ***), Single-Leg Stance Test with Eyes Open and Step Test (0.440 ***), and Sit-Up Test and Step Test (0.493 ***) were positively correlated. On the other hand, there were negative correlations between L Brain Activity and R Brain Activity (−0.593 ***), cognitive health and TUG (−0.297 *), Grip Strength and Shoulder Mobility (−0.345 **) and TUG (−0.409 **), Shoulder Mobility and Single-Leg Stance Test with Eyes Open (−0.356 **) and Step Test (−0.285 *), and TUG and Sit-Up Test (−0.306 *) and Step test (−0.325 *).

### 3.6. Effects of Physical Fitness on Cognitive Health in Older Adults

The results of the regression analysis of the effect of elderly physical fitness on cognitive health are shown in Table 7. The F value of the regression model is 2.728 *, so there is a significant effect at the *p* < 0.05 level. The coefficient of determination for the regression analysis is 0.250, which means that 25.0% of the variables used in the statistics fit the standard regression line. These results show that, among the variables of elderly physical fitness, vigour has a significant effect on cognitive health; additionally, vigour (2.609 *) has a static effect.

## 4. Discussion

Older adults with MCI were older and had significantly more chronic conditions compared to non-MCI older adults. These findings are consistent with previous research showing that advanced age and chronic disease are strongly associated with cognitive decline [1]. The higher concentration in the MCI group could be interpreted as a compensatory mechanism for maintaining cognitive performance despite cognitive decline. Similar findings have been reported in previous research on cognitive reserve and compensatory strategies in older populations [25]. Shoulder flexibility was significantly higher in the MCI group than in the non-MCI group. While this may seem counterintuitive, it may reflect the efforts of older adults with MCI to maintain or improve certain physical functions through rehabilitation programmes or physical activity. These results also suggest that MCI is a highly heterogeneous condition, where certain motor functions may be maintained despite cognitive decline [26]. Since this study focused exclusively on older women, the findings may have limited generalizability to men or individuals from diverse racial and ethnic backgrounds. Future research should aim to include broader demographic groups to enhance the applicability of the results and provide a more comprehensive understanding of MCI and its associated factors. On the other hand, non-MCI older adults had significantly higher performance in regard to the cost of living, MMSE results, handgrip strength, and open-eye standing tests. The higher cost of living in the non-MCI group suggests that better economic resources may have a positive impact on maintaining cognitive health [27]. The higher MMSE scores of the non-MCI elderly clearly demonstrate differences in cognitive function between the MCI and non-MCI groups, supporting the existing diagnostic criteria for MCI. In addition, better hand strength and balance in the non-MCI group suggests that physical activity may play an important role in maintaining cognitive function and delaying the onset of MCI. These results are consistent with the existing literature that suggests that physical activity contributes to the maintenance of cognitive function and plays an important role in delaying the onset of MCI [28].

There was a positive correlation between concentration, stress, and left brain activity. This suggests that, as concentration increases, mental workload and left brain activity tend to increase as well. In particular, the positive correlation between mental workload and left brain activity is significant because the left brain is primarily responsible for processing cognitive tasks involving logical thinking [29]. However, there was an inverse correlation between left and right brain activity, which suggests that increased left brain activation may be accompanied by a relative decrease in right brain activity. These results are consistent with previous studies related to functional asymmetries in the brain [30].

There was a positive correlation between cognitive health, handgrip strength, and step testing. This suggests that older adults with better cognitive function have better hand strength and aerobic capacity, which are indicators of physical health. These findings are consistent with existing research that suggests that physical health has a positive impact on maintaining cognitive function, specifically suggesting that overall physical activity in older adults may strengthen cognitive reserve [31]. On the other hand, there was an inverse correlation between cognitive health and the Time Up and Go (TUG) test, suggesting that mobility may be compromised as cognitive function declines. There was a positive correlation between handgrip strength and the open-eye standing test, sit-up test, and step test, but an inverse correlation between shoulder flexibility and the TUG test. This suggests that older adults with higher muscle strength are also likely to perform better in terms of balance and overall physical activity. However, the inverse correlation between muscle strength and shoulder flexibility and mobility suggests that the development of certain physical abilities may be associated with a decline in other functions [32]. There was an inverse correlation between shoulder flexibility, the open-eye standing test, and the step test, and a positive correlation with the TUG test. This suggests that higher shoulder flexibility may be associated with relatively poorer balance and aerobic performance. Conversely, higher shoulder flexibility may be associated with positive performance in mobility [33]. Positive correlations were found between the open-eye standing test and the step test and between the sit-up test and the step test, while negative correlations were found between the step test and the TUG test. This shows that balance and aerobic capacity are closely related to each other, suggesting that overall physical activity capacity may have an opposite trend to mobility [34].

This is consistent with previous research that suggests that the activity of specific brain regions may have a limited effect on cognitive function [8]. On the other hand, among the physical fitness factors in older adults, grip strength was found to have a static effect on cognitive health. This means that higher grip strength is likely to be associated with better cognitive function, suggesting that physical robustness may contribute to enhancing cognitive reserve [35]. These findings suggest that physical activity, particularly strength training, may play an important role in maintaining cognitive health in older adults [36]. However, with the exception of hand strength, all other factors of physical fitness and brain activity in older adults did not have a significant effect on cognitive health. This suggests that overall physical fitness or brain activity alone may be of limited value in preserving or improving cognitive function in older adults. This suggests that specific physical abilities, such as hand strength, may be more important in maintaining cognitive health [37]. Since this study focused exclusively on older women, the findings may have limited generalizability to men or individuals from diverse racial and ethnic backgrounds. Future research should aim to include broader demographic groups to enhance the applicability of the results and provide a more comprehensive understanding of MCI and its associated factors.

## 5. Conclusions

The findings suggest that older age and chronic disease are strongly associated with cognitive decline, and that economic resources and physical strength may play an important role in maintaining cognitive health. In particular, muscle strength, especially hand strength, was found to be a significant physical factor influencing cognitive health, suggesting that cognitive reserve can be increased by increasing muscle strength. In contrast, brain activity and other physical abilities did not have a significant effect on cognitive health, suggesting that mere physical activity or brain activity alone may be insufficient for maintaining cognitive function. Therefore, it is necessary to develop exercise programmes that focus on increasing muscle strength to maintain cognitive health in the elderly population. Furthermore, future research should further analyse the relationship between the activity of different brain regions and cognitive function and explore more comprehensive intervention strategies based on the interactions between physical ability and cognitive function. Such strategies could make an important contribution to improving the quality of life of older adults and supporting healthy ageing.

## Figures and Tables

**Table 1:** Characteristics of subjects.

Variables		N	%
MCI	MCI	32	60.71
	non-MCI	24	39.29
Age	65–75	17	30.4
	76–85	31	55.4
	Over 86	8	14.3
Education	Unacademic	10	17.9
	Elementary school	35	62.5
	Middle school and above	11	19.6
Housing type	Detached house	36	64.3
	Apartment	20	35.7
Household size	Single	23	41.1
	Two or more	33	58.9
Marital status	Married	36	64.3
	Separated, Widowed, Divorced	20	35.7
Cost of living (KRW)	1 million or less	46	82.1
	More than 1 million	10	17.9
Chronic disease	None	13	23.2
	One	19	33.9
	Two or more	24	42.9

**Table 2:** The detailed indicators of brain activity are neurophysiological EEG indices.

Variables	Neurophysiological EEG Indicators
Instant Memory	Absolute Cognitive Intensity Averaged by Difficulty Level (μV/s) Amplitude Change in Cognitive Gamma-Peak
Judgement Time	Absolute Reaction Time Averaged by Difficulty Level (s)
Concentration	(SMR + M − Beta)/Theta SMR = Sensory Motor Rhythm
Mental Workload	SEF90% (spectral edge frequency-90%)
L, R Brain Activity	Relative Left/Right Hemisphere Occurrence Ratio of Gamma Power

**Table 3:** Differences in demographics and cognitive health with and without MCI.

Variable	Non-MCI	MCI	t	95% CI Lower	95% CI Upper
Age	75.00 ± 6.51	81.41 ± 4.87	−4.044 ***	−9.61	−3.21
Education	3.25 ± 0.74	2.94 ± 0.84	1.478	−0.11	0.74
Housing type	1.46 ± 0.51	1.28 ± 0.46	1.346	−0.09	0.44
Household size	1.62 ± 0.49	1.81 ± 1.00	−0.922	−0.60	0.22
Marital status	2.25 ± 0.44	2.44 ± 0.50	−1.478	−0.44	0.07
Cost of living	100.83 ± 61.92	62.34 ± 46.85	2.547 *	7.98	69.00
Chronic disease	1.00 ± 0.83	2.06 ± 2.29	−2.424 *	−1.95	−0.18
MMSE	23.12 ± 2.58	19.06 ± 3.63	4.899 ***	2.40	5.73

M ± SD, M: Mean, SD: Standard Deviation, * *p* < 0.05, *** *p* < 0.001.

**Table 4:** Differences in the body compositions of subjects with and without MCI.

Variable	Non-MCI	MCI	t	95% CI Lower	95% CI Upper
Hight	152.46 ± 6.35	150.72 ± 7.43	0.947	−1.95	5.45
Weight	57.66 ± 8.69	58.82 ± 9.18	−0.480	−5.98	3.67
BMI(kg/m^2^)	24.74 ± 2.83	25.94 ± 3.33	−1.457	−2.85	0.45
BMR(point)	1184.17 ± 99.15	1180.00 ± 109.26	0.149	−51.97	60.30
Skeletal Muscle Mass	20.13 ± 2.69	19.97 ± 3.09	0.216	−1.39	1.72
Body Fat Mass	19.97 ± 5.25	21.32 ± 6.68	−0.847	−4.55	1.85
WHR	0.89 ± 0.05	0.92 ± 0.08	−1.709	−0.06	0.01

M ± SD, M: Mean, SD: Standard Deviation, BMI: body mass index, BMR: basal metabolic rate, WHR: waist–hip ratio.

**Table 5:** Differences in brain activity between with and without MCI.

Variable	Non-MCI	MCI	t	95% CI Lower	95% CI Upper
Instant Memory	50.00 ± 6.17	49.44 ± 5.43	0.355	−2.63	3.75
Judgement Time	33.96 ± 9.63	36.19 ± 10.35	−0.830	−7.62	3.16
Concentration	52.71 ± 14.83	63.75 ± 18.01	−2.514 *	−19.85	−2.23
Mental Workload	52.88 ± 11.69	59.53 ± 16.16	−1.788	−14.12	0.81
L Brain Activity	49.17 ± 6.54	48.56 ± 7.15	0.329	−3.09	4.29
R Brain Activity	52.29 ± 4.52	53.78 ± 4.24	−1.254	−3.88	0.90

M ± SD, M: Mean, SD: Standard Deviation, * *p* < 0.05.

**Table 6:** Differences in physical fitness between patients with and without MCI.

Variable	Non-MCI	MCI	*t*	95% CI Lower	95% CI Upper
Grip Strength	23.12 ± 6.86	18.11 ± 4.30	3.145 **	1.78	8.24
Shoulder Mobility	10.08 ± 9.85	23.16 ± 13.47	−4.197 ***	−19.33	−6.83
Single-Leg Stance Test with Eyes Open	18.71 ± 11.01	10.93 ± 10.90	2.630 *	1.84	13.73
Sit-Up Test	9.73 ± 5.04	9.06 ± 2.66	0.589	−1.64	2.97
Step Test	177.96 ± 77.10	145.09 ± 73.39	1.611	−8.14	73.87
TUG	7.87 ± 2.48	9.87 ± 5.44	−1.839	−4.19	0.19

M ± SD, M: Mean, SD: Standard Deviation, TUG: Time Up to Go, * *p* < 0.05, ** *p* < 0.01, *** *p* < 0.001.

**Table 7:** Effects of physical fitness on cognitive health in older adults.

Independent Variable	Dependent Variable	SE	*β*	*t*	VIF	df	F
Constant	Cognitive health	2.532		6.771			
Grip Strength		0.108	0.283	2.609 *	1.953		
Shoulder Mobility		0.043	−0.029	−0.673	1.546		
Single-Leg Stance Test with Eyes Open		0.048	−0.031	−0.656	1.384	6	2.728 *
Sit-Up Test		0.147	−0.069	−0.467	1.454		
Step Test		0.009	0.001	0.121	2.044		
TUG		0.129	−0.071	−0.554	1.541		

R^2^ = 0.250, Adjusted R^2^ = 0.159; TUG: Time Up to Go, * *p* < 0.05.

## Data Availability

Anonymized data will be made available to the scientific community upon reasonable request.
